# Impact of COVID-19 pandemic on the health-related quality of life of frontline workers: the case of seven low-income Eastern African countries

**DOI:** 10.1186/s12955-023-02145-7

**Published:** 2023-08-21

**Authors:** Alexis Nizigiyimana, Dilaram Acharya, Thomas G. Poder

**Affiliations:** 1 Department of Management, Evaluation and Health Policy, School of Public Health, Université de Montréal, Montréal, QC Canada; 2 Centre de REcherche en Santé Publique, Montréal, QC Canada; 3 Medical Affairs and Innovation, Héma-Québec, Montréal, QC Canada; 4 Centre Interuniversitaire de Recherche en ANalyse des Organisations, Montréal, QC Canada; 5 Centre de Recherche de l’Institut Universitaire en Santé Mentale de Montréal, CIUSSS de l’Est-de-L’île-de-Montréal, Montréal, QC Canada

**Keywords:** COVID-19, Health behaviour, Health-related quality of life, Infectious disease, Public health, Vaccination, Africa

## Abstract

**Purpose:**

This study aimed to explore the potential impact of the COVID-19 pandemic on the health-related quality of life (HRQoL) of humanitarian and healthcare workers and its related factors in seven Eastern African countries (EAC).

**Methods:**

A sample of frontline workers filled out an online cross-sectional survey questionnaire comprising socio-demographic, degree of symptoms of depression, anxiety, insomnia, and distress, alcohol and tobacco consumption, health-related quality of life (HRQoL) using Short Form 6-Dimension version 2 (SF-6Dv2) and Clinical Outcomes in Routine Evaluation 6-Dimension (CORE-6D), and fear of COVID-19 (FCV-19S) questionnaires. Multivariate regressions were conducted to identify independent factors associated with HRQoL.

**Results:**

Of total 721 study participants, mean (standard deviation) scores for SF-6Dv2 and CORE-6D were 0.87 (0.18) and 0.81 (0.14), respectively. Participants with an education level below a university degree, having chronic diseases, been tested positive to COVID-19, with traumatic memories, depression, insomnia, distress, and stress were found to have lower HRQoL likelihood in terms of SF-6Dv2 scores during the COVID-19 pandemic. Similarly, participants with chronic diseases, exposure to COVID-19 patients, depression, insomnia, distress, stress, tested positive with COVID-19, and high level of fear of COVID-19, had lower HRQoL likelihood in terms of CORE-6D scores. Participants who were married had higher HRQoL likelihoods in terms of SF-6Dv2 scores.

**Conclusion:**

Some personal and mental health characteristics, and COVID-19 related factors, were predictors of lower HRQoL of frontline workers in EAC. These findings should be meaningful while designing sustainable interventions and guidelines aiming to improve the HRQoL of frontline workers during a pandemic situation.

**Supplementary Information:**

The online version contains supplementary material available at 10.1186/s12955-023-02145-7.

## Introduction

On December 31, 2019, China reported the first COVID-19 case in Wuhan city, which subsequently spread around the world [1]. According to the latest report by the World Health Organization (WHO) in October 2022, this disease affected 220 countries, accounting for 624,639,513 confirmed cases, and 6,554,276 confirmed deaths [1]. As of March 2022, the African continent accounted for over 11 millions of COVID-19 cases, while Eastern African countries (EAC) registered more than 1.34 million cases of COVID-19, including 26,100 deaths in June 2022 [2]. WHO recommended a wide range of countermeasures, including social distancing, lockdown, personal protective measures (e.g., face masks, hand washing) to prevent and control the spread of the virus [3]. Since the beginning of this pandemic, countries have responded differently due to their differences in terms of healthcare system, sociopolitical strategies, and economic capability. For instance, Nakkazi found that Kenya, Rwanda, South Sudan and Uganda responded to COVID-19 with curfews, partial, or full lockdowns, use of personal protective measures, and social distancing, while Burundi, Tanzania and Somalia mainly opted for the use of personal protective equipment, social distancing, and hand washing without lockdowns [4]. Previous studies showed that COVID-19 diseases and associated preventive measures have adversely impacted people’s life, including frontline workers [5]. Frontline workers played a central role in the COVID-19 pandemic response, which put them at higher risks of both infections and psychological distress, fatigue and stigma [6].

According to the WHO, 80,000 to 180,000 health care workers died from COVID-19 between January 2020 and May 2021 [7]. The healthcare sector is commonly linked to elevated distress levels, which often manifest as anxiety, depression, insomnia, and burnout syndrome in its workers, even during regular circumstances. However, the SARS-CoV-2 outbreak increased the risk of healthcare workers experiencing heightened levels of stress during the pandemic due to the virus infecting a vast number of medical personnel [8, 9]. As of November 7, 2022, 23% of frontline health care workers suffered depression and anxiety and 39% suffered insomnia worldwide [10]. In a cross-sectional study among 389 Malaysian frontline healthcare workers, Woon et al. reported a lower quality of life (QoL) among health care workers with a loss of daily routine, and frequent exposure to COVID-19 patients, depression, anxiety, and stress, while those who got social support from close friends expressed higher QoL score [11]. A survey among 307 national and international humanitarian workers in Bangladesh during the COVID-19 pandemic showed a higher level of distress and, anxiety among national staff than with international staffs between July and August 2020 [12].

In a rapid review, Malizgani et al. showed that the lack of personal protective equipment, exposure to infected patients, workload, poor infection control, and pre-existing medical conditions increased the frontline workers’ risk of contracting the virus [13]. Several published studies have demonstrated a negative impact of COVID-19 on people’s mental health and health-related quality of life (HRQoL), especially for physicians, nurses, other healthcare workers, and general people of younger age group [14–18]. Recent available literature suggested that variables such as gender, age, monthly income, physical health-related factors such as comorbidities, the severity of illness, history of hospitalization due to COVID-19, mental health-related factors such as levels of stress, depression, and knowing someone infected with COVID-19 were found to be significantly associated with HRQoL of frontline workers during COVID-19 pandemic [15, 19, 20]. Clinicians, policymakers and leading organizations have recognized HRQoL as a central measure of overall health to supplement the public health’s traditional measures of morbidity and mortality [21, 22].

Several previous studies have focused on the impact of COVID-19 on mental health status, but few studies explored its impact on HRQoL, particularly among frontline healthcare workers. In a cross-sectional study conducted in Vietnam in March 2020, Than et al. showed a moderate rate of psychological distress and lower HRQoL outcomes among frontline healthcare workers during the COVID-19 outbreak [23]. In a national survey among over 10,000 Chinese frontline psychiatric clinicians, Zhang et al. found that having higher level of education, been exposed to COVID-19, being current smoker, and working overtime were significantly associated with a higher risk of depression and ultimately with a lower HRQoL [24]. Likewise, most published studies conducted in EAC explored the impact of COVID-19 on healthcare providers’ mental health. For instance, in cross-sectional studies conducted in Kenya and Ethiopia, researchers found higher rate of mental health disorders as a result of the COVID-19 pandemic [25, 26].

To the best of our knowledge, this is the first study exploring the potential impact of the COVID-19 pandemic on the HRQoL of frontline humanitarian and healthcare workers in several EAC. In this study, we examined various influential factors such as socio-demographic, availability of essential work-related supplies, COVID-19 related factors, physical and mental health related attributes to HRQoL among frontline workers in EAC. Therefore, the outcome of this study might support the respective countries’ governments, policymakers, and other stakeholders to develop evidence-based sustainable interventions and guidelines, aiming to improve the HRQoL of frontline workers, especially during the time of COVID-19 pandemic or other infectious disease pandemic situation.

## Methods

### Settings and participants

Data were collected using an online cross-sectional survey questionnaire between the 1^st^ and 20^th^ December 2020. The survey questionnaire was available in both English and French. Considering COVID-19 restrictions to minimize the spread of the pandemic, the survey was distributed via the WhatsApp platform to reach participants across EAC, namely Burundi, Rwanda, Tanzania, Somalia, South Sudan, Kenya, and Ethiopia. Previous studies suggested that the WhatsApp platform could be used to collect data given its speed and cost-effectiveness [27]. Considering that the survey was distributed via WhatsApp and the self-selected and non-probabilistic nature of the sample, the response rate was not quantifiable as aligned with the American Association for Public Opinion Research (AAPOR) reporting guideline [7].

In this study, frontline workers were defined by health care workers, including medical doctors, nurses, medical student, laboratory technicians who were working in a health facility during this pandemic, and by humanitarian workers who worked with national and international organization involved against the COVID-19 pandemic by providing a direct assistance to the population [12, 28].

### Measures

The survey questionnaire included socio-demographic variables (e.g., age, gender, education, residence, monthly income, workplace characteristics) and information regarding the direct consequences of COVID-19 such as having contact with patients or family members infected or deceased, being quarantined or infected. Frontline workers were identified as those directly involved in the prevention and control of the COVID-19 pandemic, including healthcare and humanitarian workers. Mental health disorders were classified as mild, moderate and severe, based on the degree of functional impairment on the ICD-10 classification of mental and behavioural disorders [29]. HRQoL was assessed by the Short-form Six-Dimension version 2 (SF-6Dv2) [30] and the Clinical Outcomes in Routine Evaluation Six-Dimension (CORE-6D) [31], both designed for calculating quality-adjusted life-years (QALYs), with a score of 1 for full health and 0 for death. SF-6Dv2 is an instrument derived from the SF-36v2 and assesses HRQoL on 6 dimensions: physical functioning (PF), role functioning (RF), social functioning (SF), pain (PA), mental health (MH), and vitality (VT); with 5–6 response levels each [30]. The CORE-6D is a HRQoL questionnaire dedicated to mental health which consists of 6 items, each with 5 levels of response (ranging from “not at all” to “most or all the time”), tapping 2 conceptual domains: 5 emotional items, and one physical symptom item [29]. The value sets used to calculate the scores in the QALY instrument were produced using a discrete-choice experiment (DCE) for the SF-6Dv2 [32] and the time trade-off (TTO) method for the CORE-6D [31]. Fear of COVID-19 was assessed using the French-Canadian version of the FCV-19S for French-speaking respondents [33] and the English-UK version for those responding in English [34]. In the Fear of COVID-19 scale, participants responded to seven questions by choosing: “strongly disagree,” “disagree,” “neutral, “agree,” and “strongly agree” [33] (see supplementary information (SI)—Questionnaire)).

### Statistical analysis

The collected data were entered into MS Excel and then imported into Statistical Package for the Social Sciences (SPSS), version 28 (SPSS inc., Chicago, IL). Descriptive statistics were presented to summarize frequencies and percentages for categorical variables, and the mean and standard deviation (SD), median and interquartile range for continuous variables. Univariate analyses were conducted employing non-parametric tests – Mann–Whitney U Test, or Kruskal–Wallis Test depending on the types of variables. Multivariable regression (ordinary least squares) analyses were conducted to assess the independent associations between independent HRQoL variables and the outcomes of interest. We entered all variables with p ≤ 0.1 in univariate analyses into multivariate regression analysis. The beta coefficient and odds ratio (OR) with their 95% confidence intervals (95% CI) were reported. A p-value of less than 0.05 was considered statistically significant.

### Ethical considerations

This survey was fully compliant with the indications of the Helsinki Declaration. Online consent was obtained from the participants. Participants were free to refuse to participate in the research without having to justify themselves. Failure to answer the questionnaire on their part was considered as an objection and the fact of answering the questionnaire acted as a consent. The study was approved by the ethic and research committee of the National University of Burundi in Medicine faculty (approval number: Réf.FM/CE/04/1/2021). Participation was anonymous to keep the confidentiality of participants.

## Results

### Description of study participants

Table 1 presents the personal characteristics of 721 frontline humanitarian and healthcare workers in EAC whose HRQoL was evaluated during the COVID-19 pandemic in late 2020. Based on the reports of the participants, 452/721 (62.7%), 461/721 (63.9%), and 361/717 (50.3%), were male, belonged to the 18–34 years of age group, and were married, respectively. Most of them were educated with a university degree (91.6%), urban residents (89.2%), from Burundi (82.5%), and healthcare workers (87.2%). Most of them earned $100 to $490 US dollars (47.2%), and did not have chronic disease (92.1%) or child (55.4%). Out of 269 female study participants, 5.9% were pregnant. Regarding the responses of the COVID-19 related information, most of them reported that they were tested negative to COVID-19 at the time of the survey (96.7%), were not suspected to be infected with COVID-19 (80.6%), had not been in quarantine (82.4%), were not exposed to COVID-19 in family (92.9%), or to deaths due to COVID-19 (96.0%), and most were not directly exposed to patients with COVID-19 (68.7%). More than half of the subjects (53.5%) and 30.8%, respectively, reported that they did have a shortage of personal protective equipment (PPE) and drugs during their work. The selected self-reported mental health related outcomes reported among study participants showed respectively, 142/717 (19.8%), 233/702 (33.2%), 320/720 (44.4%), 176/717 (24.6%), 239/719 (33.5%), and 430/718 (59.9%), who had traumatic memories, some form of depression, anxiety, insomnia, distress, and stress out due to the COVID-19 pandemic. Out of total participants, most (86.2%) had a score between 10–29 to the Fear of COVID-19 scale (over a maximum of 35). The mean (Standard deviation (SD)) scores of the participants for HRQoL variables such as SF-6Dv2 and CORE-6D were 0.87 (0.18) and 0.81 (0.14), respectively.

**Table 1:** Personal characteristics of the study participants in the East African Countries, 2020 (*N* = 721)

Variables	Number, N (%)
**Gender (*n* = 721)**	
Male	452 (62.7)
Female	269 (37.3)
**Age (*n* = 721)**	
18–34	461 (63.9)
35–49	237 (32.9)
≥ 50	23 (3.2)
**Marital status (*n* = 717)**	
Unmarried	356 (49.7)
Married	361 (50.3)
**Education level (*n* = 717)**	
Secondary degree or less	60 (8.4)
University degree	657 (91.6)
**Residence (*n* = 711)**	
Rural	77 (10.8)
Urban	634 (89.2)
**Country of origin (*n* = 721)**	
Burundi	595 (82.5)
Kenya	64 (8.9)
Rwanda	23 (3.2)
Somalia	5 (0.7)
South Sudan	17 (2.4)
Ethiopia	14 (1.9)
Tanzania	3 (0.4)
**Frontline workers (*n* = 721)**	
Humanitarian workers	92 (12.8)
Healthcare workers	629 (87.2)
**Type of Occupation (*n* = 721)**	
Humanitarian^a^	92 (12.8)
Medical Doctor	255 (35.4)
Medical Intern	137 (19.0)
Nurse	172 (23.9)
Social worker	28 (3.9)
Laboratory Technician	37 (5.1)
**Monthly income (*n* = 663)**	
< $100	120 (16.6)
$100-$499	340 (47.2)
≥ $500	203 (28.2)
**Chronic disease (*n* = 721)**	
No	664 (92.1)
Yes	57 (7.9)
**Currently pregnant (*n* = 269)**	
No	253 (94.1)
Yes	16 (5.9)
**Have child(ren) (*n* = 715)**	
No	396 (55.4)
Yes	319 (44.6)
**Been tested positive to COVID-19 (*n* = 721)**	
No	690 (96.7)
Yes	31 (4.3)
**Been suspected to COVID-19 (*n* = 720)**	
No	580 (80.6)
Yes	140 (19.4)
**Been in quarantine(*n* = 720)**	
No	593 (82.4)
Yes	127 (17.6)
**Been exposed to COVID-19 patients (*n* = 719)**	
No	494 (68.7)
Yes	225 (31.3)
**Been exposed to COVID-19 in family (*n* = 721)**	
No	670 (92.9)
Yes	51 (7.1)
**Been exposed to a death due to COVID-19 (*n* = 721)**	
No	692 (96.0)
Yes	29 (4.0)
**Experienced a shortage of PPE (*n* = 721)**	
No	335 (46.5)
Yes	386 (53.5)
**Experienced a shortage of drug(*n* = 720)**	
No	498 (69.2)
Yes	222 (30.8)
**Health problems due to COVID-19**	
**Been in contact with a mental health worker (*n* = 714)**	
No	663 (92.9)
Yes	51 (7.1)
**Traumatic memories (*n* = 717)**	
No	575 (79.8)
Yes	142 (19.7)
**Depressed (*n* = 702)**	
Normal	469 (66.8)
Mild	169 (24.1)
Moderate	55 (7.8)
Severe	9 (1.3)
**Anxiety (*n* = 720)**	
Normal	400 (55.6)
Mild	202 (28.0)
Moderate	101(14.0)
Severe	17 (2.4)
**Insomnia (*n* = 717)**	
Normal	541 (75.4)
Mild	124 (17.3)
Moderate	38 (5.3)
Severe	14 (2.0)
**Distressed (*n* = 719)**	
Normal	480 (66.8)
Mild	163 (22.7)
Moderate	61 (8.5)
Severe	15 (2.0)
**Stressed out (*n* = 718)**	
Normal	288 (40.1)
Mild	106 (14.8)
Moderate	238 (33.1)
Severe	86 (12.0)
**SF-6Dv2**^**b **^**(*n* = 721)**	
Mean	0.87
Std. Deviation	0.18
Minimum	0.57
Maximum	1
**CORE-6D**^**c**^** (*n* = 721)**	
Mean	0.81
Std. Deviation	0.14
Minimum	0.10
Maximum	0.95
**Fear of COVID-19**^**d**^** (*n* = 721)**	
Mean	19.27
Std. Deviation	6.42
Minimum	7.00
Maximum	35.00
**Fear of COVID-19**^**d**^** (*n* = 721)**	
0–9	63 (8.7)
10–19	296 (41.1)
20–29	325 (45.1)
More than 29	37 (5.1)

^a^Those who worked with national and international organization involved against the COVID-19 pandemic by providing a direct assistance to the population

^b^The Short Form 6-Dimension version 2 (SF-6Dv2) is a 6-dimension generic HRQoL questionnaire

^c^The Clinical Outcomes in Routine Evaluation 6-Dimension (CORE-6D) is a HRQoL questionnaire specific to mental health based on five emotional components and one physical health component

^d^The Fear of COVID-19 Score is based on the 7-item Fear of COVID-19 Scale (FCV-19S) with 5-point Likert scale from "Strongly disagree" (1) to "Strongly agree" (5). The score is ranging from 7 to 35 and corresponds to the sum of each modality

The differences in scores for SF-6Dv2 and CORE-6D by respective independent variables with their mean (SD), and median (Q3, Q1) are presented in Table 2. SF-6Dv2 and CORE-6D scores were significantly independently lower for those having traumatic memories and for some form of mental illnesses (mild to severe), such as depression, anxiety, insomnia, distress, stress out, and with those indicating high scores of fear of COVID-19 (*p* < 0.001). Similarly, the scores of SF-6Dv2 and CORE-6D were significantly independently lower for those who were tested positive, suspected of COVID-19, quarantined, exposed to COVID-19 patients, with COVID-19 cases in the family, exposed to deaths as a result of COVID-19, and experienced a shortage of PPE and drug (*p* < 0.001).

**Table 2:** Univariate analysis of factors associated with HRQoL in frontline workers in EAC, 2020 (*N* = 721)

Variables	Frequency (N)	SF-6Dv2 score			CORE-6D score		
		**Mean (SD)**	**Median (Q3, Q1)**	***P*-value**	**Mean (SD)**	**Median (Q3, Q1)**	***P*-value**
**Gender (*n* = 721)**				0.207			0.06
Male	452	0.88 (0.18)	0.91 (1.0,0.82)		0.81 (0.14)	0.87 (0.87,0.77)	
Female	269	0.85 (0.19)	0.91 (0.99,0.76)		0.79 (0.15)	0.91 (0.99,0.76)	
**Age (*n* = 721)**				0.01			0.003
18–34	461	0.88 (0.18)	0.93 (1.0,0.81)		0.81 (0.14)	0.87 (0.94,0.77)	
35–49	237	0.85 (0.19)	0.91 (0.98,0.79)		0.79 (0.15)	0.84 (0.87,0.72)	
≥ 50	23	0.86 (0.17)	0.88 (0.98,0.70)		0.81 (0.10)	0.87 (0.87,0.69)	
**Marital status (*n* = 717)**				0.012			< 0.001
Unmarried	356	0.88 (0.16)	0.94 (1.0,0.82)		0.82 (0.14)	0.87 (0.94,0.77)	
Married	361	0.86 (0.19)	0.91 (0.99,0.79)		0.79 (0.14)	0.84 (0.87,0.72)	
**Education level (*n* = 717)**				0.038			0 .854
Secondary degree or less	60	0.91(0.11)	0.97 (1.0,0.85)		0.80 (0.17)	0.87 (0.95,0.80)	
University degree	657	0.86 (0.19)	0.91 (0.99,0.80)		0.81 (0.14)	0.87 (0.87,0.73)	
**Residence (*n* = 711)**				0.101			0.002
Rural	77	0.84 (0.21)	0.88 (1.0,0.79)		0.77 (0.15)	0.80 (0.87,0.70)	
Urban	634	0.87 (0.18)	0.91 (0.99,0.80)		0.81 (0.14)	0.87 (0.87,0.77)	
**Country of origin (*n* = 721)**				< 0.001			< 0.001
Burundi	595	0.89 (0.15)	0.95 (1.0, 0.85)		0.81 (0.14)	0.87 (0.71,0.15	
Kenya	64	0.70 (0.24)	0.74(0.89, 0.56)		0.73 (0.12)	0.77 (0.63,0.17)	
Rwanda	23	0.86(0.12)	0.87(1.0, 0.78)		0.81(0.13)	0.87(0.45,0.22)	
Somalia	5	0.99 (0.70)	0.99 (0.99, 0.98)		0.68 (0.33)	0.87 (0.77,0.46)	
South Sudan	17	0.77(0.34)	0.84 (0.96, 0.76)		0.76 (0.18)	0.80 (0.84, 0.17)	
Ethiopia	14	0.76 (0.21)	.81 (0.93, 0.67)		0.74 (0.10)	0.77 (0.37,0.10)	
Tanzania	3	0.87 (0.06)	0.83 (0.11,0.001)		0.83 (0.05)	0.87 (0.10,0.00)	
**Frontline workers (*n* = 721)**				0.56			0.002
Humanitarian	92	0.86 (0.18)	0.92 (0.99,0.77)		0.81(0.16)	0.87 (0.91,0.77)	
Healthcare worker	629	0.87 (0.18)	0.91 (1.00,0.80)		0.80 (0.14)	0.87 (0.87,0.73)	
**Type of occupation**				0.18			0.08
Humanitarian	92	0.88 (0.12)	0.91 (0.33,1.00)		0.82 (0.13)	0.87 (0.32,0.95)	
Medical Doctor	255	0.85 (0.19)	0.91 (0.57,1.00)		0.78 (0.14)	0.84 (0.1,0.95)	
Medical Intern	137	0.88 (0.15)	0.95 (0.21,1.00)		0.81 (0.13)	0.87 (0.24,0.95)	
Nurse	172	0.86 (0.21)	0.94 (0.47,1.00)		0.81 (0.15)	0.87 (0.10,0.95)	
Social worker	28	0.86 (0.15)	0.93 (0.44,10.00)		0.79 (0.11)	0.82 (0.58,0.95)	
Laboratory Technician	37	0.88 (0.18)	0.96 (0.18,1.00)		0.80 (0.15)	0.87 (0.41,0.95)	
**Monthly income (*n* = 663)**				0.01			< 0.001
< $100	120	0.90 (0.16)	0.96 (1.00,0.73)		0.85 (0.10)	0.87 (0.94,0.80)	
$100-$4909	340	0.87 (0.16)	0.91 (1.00,0.80)		0.80 (0.14)	0.84 (0.87,0.72)	
≥ $500	203	0.84 (0.22)	0.90 (1.00,0.74)		0.80 (0.15)	0.86 (0.87,0.77)	
**Chronic disease (*n* = 721)**				< 0.001			0.003
No	664	0.87 (0.18)	0.92 (1.00,0.81)		0.81 (0.14)	0.87 (0.87,0.77)	
Yes	57	0.80 (0.25)	0.81 (0.96,0.74)		0.75 (0.17)	0.80 (0.87,0.69)	
**Currently pregnant (*n* = 269)**				0.55			0.220
No	253	0.86 (0.19)	0.93(1.00,0.79)		0.80 (0.15)	0.84 (0.87,0.72)	
Yes	16	0.81 (0.27)	0.91 (0.98,0.73)		0.77 (0.11)	0.79 (0.87,0.70)	
**Have child(ren) (*n* = 715)**				0.01			< 0.001
No	396	0.88 (0.18)	0.94 (1.00,0.82)		0.87 (0.18)	0.87 (0.94,0.77)	
Yes	319	0.85 (0.19)	0.91(0.99,0.77)		0.82 (0.14)	0.80 (0.87,0.72)	
**Been tested positive to COVID-19 (*n* = 721)**				0.01			< 0.001
No	690	0.87 (0.17)	0.91 (1.00,0.80)		0.87 (0.18)	0.87 (0.87,0.77)	
Yes	31	0.75 (0.36)	0.85 (0.96,0.74)		0.81 (0.14)	0.72 (0.87,0.69)	
**Been suspected to COVID-19 (*n* = 720)**				< 0.001			< 0.001
No	580	0.89 (0.15)	0.95 (1.00,0.83)		0.82 (0.14)	0.87 (0.90,0.77)	
Yes	140	0.76 (0.27)	0.83 (1.00,0.67)		0.75 (0.16)	0.77 (0.87,0.69)	
**Been in quarantine (*n *= 720)**				< 0.001			< 0.001
No	593	0.89 (0.15)	0.95 (1.00,0.83)		0.82 (0.14)	0.87 (0.90,0.77)	
Yes	127	0.76 (0.27)	0.82 (1.00,0.82)		0.75 (0.16)	0.77 (0.87,0.69)	
**Been exposed to COVID-19 patients (*n* = 719)**				< 0.001			< 0.001
No	494	0.90(0.15)	0.96 (1.00,0.85)		0.83(0.13)	0.87 (0.92,0.80)	
Yes	225	0.80(0.23)	0.84 (0.96,0.69)		0.76(0.16)	0.80 (0.87,0.69)	
**Been exposed to COVID-19 in family (*n* = 721)**				< 0.001			< 0.001
No	670	0.88 (0.17)	0.92 (1.00,0.82)		0.81 (0.14)	0.87 (0.87,0.77)	
Yes	51	0.75 (0.31)	0.81 (0.92,0.65)		0.74(0.18)	0.77 (0.87,0.65)	
**Been exposed to a death due to COVID-19 (*n* = 721)**				0.001			< 0.001
No	692	0.88 (0.17)	0.92 (1.00,0.84)		0.81 (0.14)	0.87 (0.87,0.77)	
Yes	29	0.70 (0.39)	0.79 (0.92,0.65)		0.67 (0.21)	0.73 (0.81,0.61)	
**Experienced a shortage of PPE (*n* = 721)**				< 0.001			< 0.001
No	335	0.90 (0.14)	0.96 (1.00,0.84)		0.82 (0.14)	0.87 (0.94,0.79)	
Yes	386	0.84 (0.21)	0.89 (1.00,0.74)		0.79 (0.15)	0.84 (0.87,0.72)	
**Experienced a shortage of drug (*n* = 720)**				< 0.001			< 0.001
No	498	0.90 (0.15)	0.95 (1.00,0.83)		0.82 (0.14)	0.87 (0.94,0.77)	
Yes	222	0.81(0.23	0.86 (0.96,0.70)		0.77 (0.16)	0.80 (0.87,0.69)	
**Having contacted a mental health worker (*n* = 721)**				< 0.001			< 0.001
No	663	0.88 (0.16)	0.92 (1.00,0.82)		0.81 (0.14)	0.87 (0.87,0.77)	
Yes	51	0.74 (0.30)	0.81 (0.91,0.65)		0.74 (0.18)	0.77 (0.87,0.61)	
**Health problems due to COVID-19**							
**Traumatic memories (*n* = 717)**				< 0.001			< 0.001
No	575	0.90 (0.13)	0.95 (1.00,0.83)		0.82 (0.13)	0.87 (0.90,0.77)	
Yes	142	0.75(0.27)	0.84 (0.92,0.64)		0.74(0.17)	0.77 (0.87,0.65)	
**Depressed (*n* = 702)**				< 0.001			< 0.001
Normal	469	0.91(0.13)	0.96 (1.00,0.88)		0.84 (0.12)	0.87 (0.94,0.80)	
Mild	169	0.82 (0.17)	0.83 (0.95,0.71)		0.77 (0.14)	0.80 (0.87,0.69)	
Moderate	55	0.71(0.22)	0.77 (0.87,0.52)		0.71(0.17)	0.77 (0.87,0.61)	
Severe	9	0.36 (0.41)	0.34 (0.63,0.22)		0.53 (0.24)	0.50 (0.75,0.37)	
**Anxiety (*n* = 720)**				< 0.001			< 0.001
Normal	400	0.92 (0.13)	0.97 (1.00,0.88)		0.84(0.12)	0.87 (0.94,0.80)	
Mild	202	0.85(0.16)	0.90 (0.96,0.77)		0.80(0.13)	0.84 (0.87,0.77)	
Moderate	101	0.76(0.20)	0.79 (0.88,0.66)		0.73(0.16)	0.77 (0.87,0.64)	
Severe	17	0.56(0.41)					
**Insomnia (*n* = 717)**				< 0.001			< 0.001
Normal	541	0.91(0.12)	0.96 (1.00,0.87)		0.83(0.12)	0.87 (0.94,0.80)	
Mild	124	0.78 (0.18)	0.80 (0.91,0.66)		0.75(0.15)	0.77 (0.87,0.66)	
Moderate	38	0.67 (0.26)	0.78 (0.88,0.52)		0.68 (0.18)	0.71 (0.86,0.63)	
Severe	14	0.51(0.40)	0.37 (0.69,0.32)		0.59 (0.21)	0.61 (0.70,0.45)	
**Distressed (*n *= 719)**				< 0.001			< 0.001
Normal	480	0.92 (0.12)	0.96 (1.00, 0.88)		0.84 (0.12)	0.87 (0.94, 0.80)	
Mild	163	0.83 (0.17)	0.86 (0.95,0.73)		0.78 (0.13)	0.80 (0.87,0.72)	
Moderate	61	0.67(0.24)	0.66 (0.80,0.45)		0.66(0.19)	0.64 (0.77,0.52)	
Severe	15	0.55 (0.40)	0.54 (0.77,0.21)		0.63 (0.20)	0.65 (0.77,0.52)	
**Stressed out (*n* = 718)**				< 0.001			< 0.001
Normal	288	0.94 (0.09)	0.99 (1,0.91)		0.86 (0.10)	0.87 (0.94,0.84)	
Mild	106	0.87(0.16)	0.91 (0.96,0.79)		0.84 (0.09)	0.87 (0.87,0.80)	
Moderate	238	0.84 (0.18)	0.87 (0.96,0.76)		0.77 (0.15)	0.80 (0.87,0.69)	
Severe	86	0.72(0.26)	0.73 (0.88,0.52)		0.69 (0.17)	0.69 (0.80,0.52)	
**Fear of COVID-19 (*n* = 721) **				< 0.001			< 0.001
0–9	63	0.91(0.14)	0.98 (0.81;0.12)		0.85 (0.10)	0.87 (0.54;0.08)	
10–19	296	0.88(0.15)	0.92(1.2;0.16)		0.82(0.10)	0.87(0.63;0.10)	
20–29	325	0.86 (0.16)	0.91(1.1;0.20)		0.79(0.15)	0.84 (0.71;0.17)	
> 29	37	0.66 (0.40)	0.80 (1.5;0.56)		0.66 (0.23)	0.69 (0.85;0.27)	

The scores of SF-6Dv2 also varied with other background characteristics such as age, marital status, education, residence, country of origin, and monthly income (*p* < 0.05), while scores of CORE-6D significantly differed by background characteristics such as age, marital status, residence, country of origin, occupation, and income (*p* < 0.05). About 54.4% responded in French language while 45.6% responded in English language.

### Factors associated with HRQoL (SF-6Dv2 and CORE-6D) in frontline workers by multivariate analyses

In multivariate analyses, several influential factors associated with lower HRQoL were observed (Table 3). Participants with an education level below a university degree (OR = 0.97, 95% CI: 0.92–0.99), having a chronic disease (OR = 0.95, 95% CI: 0.91–0.99), been tested positive with COVID-19 (OR = 0.94, 95% CI: 0.91–0.96), traumatic memories (OR = 0.96, 95% CI: 0.93–0.99), depression (OR = 0.95, 95% CI: 0.93–0.97), insomnia (OR = 0.96, 95% CI: 0.93–0.98), distress (OR = 0.95, 95% CI: 0.93–0.97), stress out (OR = 0.98, 95% CI: 0.97–0.99) due to COVID-19, were found to have a lower HRQoL likelihood in terms of SF-6Dv2 scores during COVID-19 pandemic. Similarly, study subjects with chronic diseases (OR = 0.97, 95% CI: 0.93–0.99), exposure to COVID-19 patients (OR = 0.98, 95% CI: 0.96–0.99), depression (OR = 0.98, 95% CI: 0.96–0.99), insomnia (OR = 0.98, 95% CI: 0.95–0.99), distress (OR = 0.98, 95% CI: 0.67–0.99), stress out (OR = 0.98, 95% CI: 0.90–0.97), and high fear of COVID-19 (OR = 0.99, 95% CI: 0.96–0.99), had lower HRQoL likelihoods in terms of CORE-6D. To the contrary, participants who were married (OR = 1.02, 95% CI: 1.003–1.04) had higher HRQoL likelihoods in terms of SF-6Dv2 scores.

**Table 3:** Factors associated with HRQoL (in terms of SF-6Dv2 and CORE-6D scores) in frontline workers in EAC, 2020 by multivariate regression analysis

**Variables**	**SF-6Dv2 score**			**CORE-6D score**		
	B	OR (95%CI)	*P*-value	B	OR (95%CI)	*P*-value
Marital status (ref. unmarried)	0.02	1.02 (1.003–1.04)	0.03	-	-	-
Education level (ref. university degree)	-0.04	0.97 (0.92–0.99)	0.04	-	-	-
Chronic disease (ref. no)	-0.05	0.95 (0.91–0.99)	0.006	-0.03	0.97 (0.93–0.99)	0.03
Country of origin (ref. Burundi)				0.007	0.93 (0.99–1.01)	0.08
Been tested positive to COVID-19 (ref. yes)	-0.06	0.94 (0.91–0.96)	0.04			
Been exposed to COVID-19 patients (ref. no)	-	-		-0.02	0.98 (0.96–0.99)	0.02
Been exposed to a death due to COVID-19 (ref. no)	-	-		-0.04	0.96 (0.92–1.01)	0.08
Traumatic memories (ref. no)	-0.04	0.96 (0.93–0.99)	< 0.001	-	-	-
Depressed (ref. normal)	-0.05	0.95 (0.93–0.97)	< 0.001	-0.02	0.98 (0.96–0.99)	0.01
Insomnia (ref. normal)	-0.04	0.96 (0.93–0.98)	< 0.001	-0.02	0.98 (0.95–0.99)	0.04
Distressed (ref. normal)	-0.05	0.95 (0.93–0.97)	< 0.001	-0.02	0.98 (0.67–0.99)	0.001
Stressed out (ref. normal)	-0.02	0.98 (0.97–0.99)	< 0.001	-0.02	0.98 (0.90–0.97)	< 0.001
Fear of COVID-19 (ref FCV19 SCORE > 29)	-	-	-	-0.003	0.99 (0.96–0.99)	< 0.001

## Discussion

Frontline workers have been recognized as the backbone to fight against SARS-CoV-2 infections worldwide but are at higher risk of infection and mortality during the COVID-19 pandemic situation [35, 36]. The COVID-19 pandemic has exposed frontline workers such as healthcare workers to a high level of vulnerability, emphasizing the critical need to provide immediate and optimal support to preserve their well-being and prevent potentially catastrophic mental health outcomes [37]. This study identified the impact of the COVID-19 pandemic on HRQoL among frontline workers and some associated factors for the first time in seven EAC. Among the multiple HRQoL questionnaires used during the pandemic, such as SF-36 [38], WHOQOL-BREF [19, 24, 39], SF-12v2) [40], EQ-5D-5L [23], we found some studies that used the SF-6D and CORE-6D questionnaires to assess HRQoL in the general population, but none for frontline workers in EAC [41, 42]. Although there is report of consistent outcome of interest between HRQoL instruments measuring same or similar dimensions of ill-health at population level, variability in the use of test instruments and different health dimension measurements in population-based study could hamper the comparability of HRQoL [43, 44]. For example, SF-6D derived from SF-36 demonstrated higher discriminative power for physical and mental health dimension than SF-12 and it is therefore, suggested to use when the population based self-reported health index is needed [44]. A similar study from Jordan reported that physicians' level of HRQoL was relatively lower during COVID-19 pandemic measured by SF-12 mental and physical components, physicians' evaluation of work conditions during COVID-19 pandemic, Neck Disability Index (NDI), Depression Anxiety Stress Scale (DASS 21), and International Physical Activity Questionnaire (IPAQ). Contrary to this study, we found that our HRQoL score measured through SF-6Dv2 was relatively higher. Such contrasting findings between the Jordanian and our study might be because of different study contexts such as type of study participants, test instruments variation, and time of study among others. This study was conducted after eight months of pandemic in seven EAC with different health system context, COVID-19 response strategies and number of confirmed cases [45–51]. For instance, this study was conducted when Burundi, Tanzania, and Somalia accounted few cases with no lockdown measures while other high, middle, and low-income-countries already applied lockdown with other restrictions, and this may explain the difference between our study results with other countries [46, 50–52]

We also found that the score variations among EAC with higher score of HRQoL in both Burundi and Tanzania compared to other EAC in an unadjusted estimate. Although the country of origin became non-significant as a determinant of HRQoL in adjusted estimates, this difference could be explained by different factors such as different number of COVID-19 cases, COVID-19 preventives measures implemented, political and socioeconomic factors in EAC [45, 47, 48, 50, 51, 53, 54]. Also, the fact that the majority responded in French language (54.4%) compared to those who responded in English language (45.6%) may explain differences as previous studies have demonstrated that HRQol differed by language and culture [55]. Even if we used the same value set for each QALY instrument, how questions were formulated may indeed be understood in a different way in different languages.

Previous studies from Italy [17], China [24], Vietnam [23], and Egypt [56] reported several mental health problems such as depression, anxiety, distress, and stress occurred in healthcare workers during the COVID-19 pandemic situation with lower rates of these problems compared to our study. This difference could be attributable to variations in participants’ background characteristics, geography, study period, and other frontline workers’ work-related factors such as availability of protective measures during work, work load and level of work satisfaction [36].

Our results in multivariate analyses indicated that lower educational achievement was significantly positively associated with a lower score of HRQoL in terms of SF-6Dv2 score, while presence of chronic diseases was significantly positively associated with a lower HRQoL likelihoods (both in terms of SF-6Dv2 and CORE-6D scores). We assume our study participants who were with a higher education level might have had higher knowledge about COVID-19 that led to lower mental stress, higher financial resources and living condition than their lower educated counterparts resulting into higher HRQoL. A wide range of literature [57–59] supports the idea that a higher level of education improves the quality of life and overall population health. The presence of chronic health condition is a negative correlates of poor HRQoL [60]. This seems true in case of COVID-19 as well. A study from Vietnam found a lower HRQoL among frontline workers who had chronic conditions during the COVID-19 pandemic [23]. Studies suggest that subjects with chronic diseases are prone to have poor mental health status [61], and higher risk of mortality from COVID-19 [62], which could subsequently result in decreased HRQoL.

Given that healthcare workers are frequently exposed to highly contagious illnesses like COVID-19, they are at a greater risk of becoming infected, which can lead to an increase in their levels of stress, depression, and anxiety [9]. We observed that several mental health attributes and COVID-19 related variables were positively associated with the HRQoL of frontline workers. Traumatic memories, depression, insomnia, distress, and stress were found to have a lower HRQoL likelihood in terms of SF-6Dv2 scores, while depression, insomnia, distress, stress, exposure to COVID-19 patients, and fear of COVID-19 had a lower HRQoL likelihood in terms of CORE-6D. Numerous studies around the world are in line with the findings of our study [15, 23, 24, 52, 56, 62, 63]. For instance, in a study carried out among 173 healthcare workers (HCW) in two national tertiary hospitals in Vietnam, Hung et al. reported a lower score of HRQoL among HCW with mental health issues (depression, stress, and anxiety) than their counterparts [23]. Another national survey conducted among frontline clinicians in China found a lower HRQoL among those who were depressed [24]. Throughout the COVID-19 pandemic situation, whether it was in resourceful or resource-limited settings, frontline workers were confronted with a fear of COVID-19, overload, and shortage of PPE [35, 36, 64]. These reasons should have placed frontline workers in a stressful situation impacting on poor HRQoL. Specific strategies such as informational, instrumental, organizational, emotional, and psychological supports provided to frontline workers could prevent or decrease short-term and long-term impact of pandemic on mental health and improve HRQoL of frontline workers [65].

We also found that frontline workers who were exposed to COVID-19 expressed a lower score of HRQoL than their non-exposed counterparts. Previously published studies demonstrated that people exposed to COVID-19 had a higher risk of psychological problems (depression, anxiety, insomnia, and stress), which might explain the reason behind the lower score of HRQoL [66]. The pandemic has placed frontline workers in a vulnerable situation of infection or death from COVID-19. This situation did not only restrict their social relationship with their relatives and neighbours, but also reduced their physical capability, and that might have ultimately lowered their HRQoL [67]. Likewise, frontline workers who tested positive in our study were found to have a lower score of HRQoL. In research conducted among HCW in Bangladesh, Rahman et al. showed a lower score of HRQoL among HCW who were infected, but those who recovered demonstrated a little improvement in HRQoL score [20]. Isolation and being under heavy stress of spreading the virus to their family members, children and neighbors, may explain the lower score of HRQoL among HCW who were tested positive. It was also argued that recovery reduced the stress and fear of being infected again due to immunity obtained, which may explain the improvement of HRQoL among recovered HCW in Bangladesh [20].

Our results were generally in line with most of published studies for a significant adverse impact of fear of COVID-19 on mental health and HRQoL [68, 69]. Several recent articles have stated that the COVID-19 pandemic has caused healthcare workers worldwide, especially in African countries, to experience fear, resulting in a reduced quality of life [70–72]. Nonetheless, one study found that paramedic students in Norway reported a higher-than-average quality of life four months into the pandemic's first wave. However, the same category of healthcare workers experienced a decline in quality of life during the third wave of the pandemic [73], indicating that all frontline healthcare providers need to be closely monitored and receive targeted interventions during pandemics.

Our study findings also showed a higher score of HRQoL among married frontline workers. Prior similar studies yielded a conflicting result on the relationship between marital status and HRQoL. For example, in a multicentric cross‑sectional survey among Indian nurses, Sharma et al. found that nurses who were married had lower HRQoL scores [74] while Han et al. reported that married people had higher HRQoL than other marital status (single, divorced) but this relationship changed when they considered the age group, where married men under the age of 30 years did not have better HRQoL than non-married peers [75]. We may argue that cultural differences between countries or communities might have contributed to such contrasting results.

The results of this study should be considered in light of some limitations. First, since we adopted an online survey methodology, we could not estimate the population distribution, poor compliance of responses for different sets of queries and control over possible sample contamination [76]. Second, our outcome of interest is related to only seven EAC that lack generalizability of study findings beyond the settings. Thirdly, some of our study participants may have faced difficulties in responding to our questionnaire due to their limited proficiency in English or French languages, which could have resulted in a language barrier and distorted the outcome of our study [77]. However, it should also be considered that English or French are the official languages taught in educational institutions in these countries. Despite of these limitations, it has some notable strengths. First, this study used validated HRQoL questionnaires internationally recognized in assessing the HRQoL with a good discriminative power compared to others. In addition, the sample size was relatively large enough to provide informative results. These study findings might be useful in benchmark for designing sustainable interventions and guidelines aiming to improve the HRQoL of frontline workers during the pandemic situation.

## Conclusions

This study found that the mean (SD) score of HRQoL of study participants for SF-6Dv2 and CORE-6D, respectively, were of 0.87 (0.18) and 0.81 (0.14). In addition, some participants’ personal attributes such as lower educational achievement, having chronic diseases, tested positive to COVID-19, mental health characteristics such as traumatic memories, depression, insomnia, distress, and stress, COVID-19 related factors such as fear of COVID-19, and exposure to COVID-19 patients, were negatively related to HRQoL, while those who were married had higher HRQoL likelihoods in terms of SF-6Dv2 scores. These findings should be sought while designing sustainable interventions and guidelines aiming to improve the HRQoL of frontline workers, especially during COVID-19 pandemic situation or other infectious disease pandemic conditions in EAC and similar settings.

### Supplementary Information

**Additional file 1:** Supplementary Information (SI): Questionnaire Survey.
