# Supplementary material for: Impact of COVID-19 pandemic on the health-related quality of life of frontline workers: the case of seven low-income Eastern African countries
Source: Health Qual Life Outcomes. 2023 Aug 21;21:97. doi: 10.1186/s12955-023-02145-7 (PMC10463627; doi:10.1186/s12955-023-02145-7)
Supplement: Supplementary file 1 — Additional file 1: Supplementary Information (SI): Questionnaire Survey. [file 12955_2023_2145_MOESM1_ESM.docx]

**Supplementary Information (SI): Questionnaire Survey**

| **1. Socio-demographic questions** | | |
| --- | --- | --- |
| SN |  |  |
| 1.1 | Gender |  |
|  |  |  |
|  |  | Men |
|  |  | Women |
| 1.2 | Age (in years) |  |
|  |  | 18-24 |
|  |  | 25-29 |
|  |  | 30-34 |
|  |  | 35-39 |
|  |  | 40-44 |
|  |  | 45-49 |
|  |  | ≥50 |
| 1.3 | Marital status |  |
|  |  | Married |
|  |  | Unmarried |
| 1.4 | Education |  |
|  |  | Primary |
|  |  | Secondary |
|  |  | University |
| 1.5 | Residence |  |
|  |  | Rural |
|  |  | Urban |
| 1.6 | Occupation |  |
|  |  | Medical doctor |
|  |  | Nurse |
|  |  | Social worker |
|  |  | Pharmacist |
|  |  | Laboratory technician |
|  |  | Medical intern |
|  |  | Humanitarian worker |
| 1.7 | Monthly income range (in USD) |  |
|  |  | <100$ |
|  |  | 100-499$ |
|  |  | ≥500$ |
|  |  | Others, indicate: |
| 1.8 | Type of hospital or humanitarian NGO |  |
|  |  | Tertiary Hospital Centre (e.g., Kamenge University Hospital, Bujumbura Hospital Prince Regent Charge, Clinic Prince Louis Rwagasore) |
|  |  | Regional hospital |
|  |  | District hospital |
|  |  | Private hospital |
|  |  | Primary health center |
|  |  | Rapid response team |
|  |  | Humanitarian NGO |
| 1.9 | Type of district | Indicate |
| 1.10 | Humanitarian NGO | Indicate |
| **2. Medical and family history-related questions** | | |
| 2.1 | Do you have a chronic disease(s)? |  |
|  |  | Yes |
|  |  | No |
| 2.2 | If yes, which is or are of these? |  |
|  |  | Diabetes |
|  |  | Hypertension |
|  |  | Cancer |
|  |  | Others (indicate) |
| 2.3 | Are you pregnant (for married women of reproductive age group only)? |  |
|  |  | Yes |
|  |  | No |
| 2.4 | Do you have a child(ren)? |  |
|  |  | Yes |
|  |  | No |
| **3. COVID-19 related general questions** | | |
| 3.1 | Have you been tested positive for COVID-19? |  |
|  |  | Yes |
|  |  | No |
| 3.2 | Have you been suspected of COVID-19? |  |
|  |  | Yes |
|  |  | No |
| 3.3 | Have you been in quarantine or self-isolated at home? |  |
|  |  | Yes |
|  |  | No |
| 3.4 | Have you been exposed to a positive COVID-19 patient? |  |
|  |  | Yes |
|  |  | No |
| 3.5 | Have you been exposed to a COVID-19 case in your family/house? |  |
|  |  | Yes |
|  |  | No |
| 3.6 | Have you been exposed to a death related to the epidemic in your family/house? |  |
|  |  | Yes |
|  |  | No |
| 3.7 | Have you experienced a shortage of personal protective equipment (PPE) (face mask, gloves etc.) in your health facility or NGO due to COVID-19? |  |
|  |  | Yes |
|  |  | No |
| 3.8 | Have you experienced shortage of medicine (medicament/drug) due to COVID-19? |  |
|  |  | Yes |
|  |  | No |
| **4. Impact of COVID-19 on mental health** | | |
| 4.1 | Have you contacted a health professional for mental health reasons since the beginning of the pandemic? |  |
|  |  | Yes |
|  |  | No |
| 4.2 | Has the current situation related to the epidemic actively brought back (flashbacks, nightmares, etc.) one or more traumatic memories that you may have experienced in the past (death, illness, panic, physical or sexual assault, etc.)? |  |
|  |  | Yes |
|  |  | No |
| 4.3 | Have you been stigmatized due to COVID-19? |  |
|  |  | Yes |
|  |  | No |
| 4.4 | Have you experienced depression symptom related to COVID-19 pandemic at workplace or at home |  |
|  |  | Normal |
|  |  | Mild |
|  |  | Moderate |
|  |  | Severe |
| 4.5 | Have you experienced anxiety symptom related to COVID-19 pandemic at workplace or at home? |  |
|  |  | Normal |
|  |  | Mild |
|  |  | Moderate |
|  |  | Severe |
| 4.6 | Have you experienced insomnia symptom (difficulties to sleep) related to COVID-19 pandemic at workplace or at home? |  |
|  |  | Normal |
|  |  | Mild |
|  |  | Moderate |
|  |  | Severe |
| 4.7 | Have you experienced distress symptom (anxiety, depression, anger, fatigue, nervousness, back pain or headache) related to COVID-19 pandemic at workplace or at home? |  |
|  |  | Normal |
|  |  | Mild |
|  |  | Moderate |
|  |  | Severe |
| 4.8 | Did you feel constantly tense or "stressed out"? |  |
|  |  | Not at all |
|  |  | No more than usual |
|  |  | A little more than usual |
|  |  | Much more than usual |
| 4.9 | Have you changed your alcohol consumption since the pandemic? |  |
|  |  | Do not drink alcohol |
|  |  | No |
|  |  | Moderate increase |
|  |  | Significant increase |
|  |  | Moderate decrease |
|  |  | Significant decrease |
| 4.10 | Have you changed your tobacco consumption since the pandemic? |  |
|  |  | I do not smoke |
|  |  | No |
|  |  | Moderate increase |
|  |  | Significant increase |
|  |  | Moderate decrease |
|  |  | Significant decrease |
| **5. The next six questions focus on different aspects of physical and mental health. Please answer each question by choosing the answer that best describes your own health status (*SF-6Dv2*).** | | |
| 5.1 | Does your health now limit you in your daily physical activities? |  |
|  |  | Not limited at all in vigorous activities (such as running, lifting heavy objects, participating in strenuous sports) |
|  |  | Limited a little in vigorous activities (such as running, lifting heavy objects, participating in strenuous sports) |
|  |  | Limited a little in moderate activities (such as moving a table, pushing a vacuum cleaner, bowling, or playing golf) |
|  |  | Limited a lot in moderate activities (such as moving a table, pushing a vacuum cleaner, bowling, or playing golf) |
|  |  | Limited a lot in bathing and dressing yourself |
| 5.2 | During the past 4 weeks, how much of the time have you accomplished less than you would like in your work or other regular daily activities as a result of your physical health or of any emotional problems (such as feeling depressed or anxious)? |  |
|  |  | None of the time |
|  |  | A little of the time |
|  |  | Some of the time |
|  |  | Most of the time |
|  |  | All of the time |
| 5.3 | How much bodily pain have you had during the past 4 weeks? |  |
|  |  | No pain |
|  |  | Very mild pain |
|  |  | Mild pain |
|  |  | Moderate pain |
|  |  | Severe pain |
|  |  | Very severe pain |
| 5.4 | How much of the time during the past 4 weeks did you feel worn out? |  |
|  |  | None of the time |
|  |  | A little of the time |
|  |  | Some of the time |
|  |  | Most of the time |
|  |  | All of the time |
| 5.5 | During the past 4 weeks, how much of the time has your physical health or emotional problems interfered with your social activities (like visiting with friends, relatives, etc.)? |  |
|  |  | None of the time |
|  |  | A little of the time |
|  |  | Some of the time |
|  |  | Most of the time |
|  |  | All of the time |
| 5.6 | How much of the time during the past 4 weeks have you felt downhearted and depressed or very nervous? |  |
|  |  | None of the time |
|  |  | A little of the time |
|  |  | Some of the time |
|  |  | Most of the time |
|  |  | All of the time |
| **6. Please read each statement and think how often you felt that way last week.** | | |
| 6.1 | I have felt terribly alone and isolated |  |
|  |  | Not at all |
|  |  | Only occasionally |
|  |  | Sometimes |
|  |  | Often |
|  |  | Most or all the time |
| 6.2 | I have felt panic or terror |  |
|  |  | Not at all |
|  |  | Only occasionally |
|  |  | Sometimes |
|  |  | Often |
|  |  | Most or all the time |
| 6.3 | I have felt humiliated or shamed by other people |  |
|  |  | Not at all |
|  |  | Only occasionally |
|  |  | Sometimes |
|  |  | Often |
|  |  | Most or all the time |
| 6.4 | I have been able to do most things I needed to |  |
|  |  | Not at all |
|  |  | Only occasionally |
|  |  | Sometimes |
|  |  | Often |
|  |  | Most or all the time |
| 6.5 | I made plans to end my life |  |
|  |  | Not at all |
|  |  | Only occasionally |
|  |  | Sometimes |
|  |  | Often |
|  |  | Most or all the time |
| 6.6 | I have been troubled by aches, pains or other physical problems |  |
|  |  | Not at all |
|  |  | Only occasionally |
|  |  | Sometimes |
|  |  | Often |
|  |  | Most or all the time |
| **7. Questions about the Fear of COVID-19 (Please respond to each item by ticking one of the five (5) responses that reflects how you feel, think or act toward COVID-19)** | | |
| 7.1 | I am most afraid of Corona |  |
|  |  | Strongly disagree |
|  |  | Disagree |
|  |  | Neutral |
|  |  | Agree |
|  |  | Strongly agree |
| 7.2 | It makes me uncomfortable to think about Corona |  |
|  |  | Strongly disagree |
|  |  | Disagree |
|  |  | Neutral |
|  |  | Agree |
|  |  | Strongly agree |
| 7.3 | My hands become clammy when I think about Corona |  |
|  |  | Strongly disagree |
|  |  | Disagree |
|  |  | Neutral |
|  |  | Agree |
|  |  | Strongly agree |
| 7.4 | I am afraid of losing my life because of Corona |  |
|  |  | Strongly disagree |
|  |  | Disagree |
|  |  | Neutral |
|  |  | Agree |
|  |  | Strongly agree |
| 7.5 | When I watch news and stories about Corona on social media, I become nervous or anxious |  |
|  |  | Strongly disagree |
|  |  | Disagree |
|  |  | Neutral |
|  |  | Agree |
|  |  | Strongly agree |
| 7.6 | I cannot sleep because I’m worrying about getting Corona |  |
|  |  | Strongly disagree |
|  |  | Disagree |
|  |  | Neutral |
|  |  | Agree |
|  |  | Strongly agree |
| 7.7 | My heart races or palpitates when I think about getting Corona |  |
|  |  | Strongly disagree |
|  |  | Disagree |
|  |  | Neutral |
|  |  | Agree |
|  |  | Strongly agree |

Thank you very much for your participation in the survey.
